# Multi‐objective evolutionary optimization for hardware‐aware neural network pruning

**DOI:** 10.1016/j.fmre.2022.07.013

**Published:** 2022-08-09

**Authors:** Wenjing Hong, Guiying Li, Shengcai Liu, Peng Yang, Ke Tang

**Affiliations:** a Guangdong Provincial Key Laboratory of Brain-Inspired Intelligent Computation, Department of Computer Science and Engineering, Southern University of Science and Technology, Shenzhen 518055, China; b Research Institute of Trustworthy Autonomous Systems, Southern University of Science and Technology, Shenzhen 518055, China; c Department of Statistics and Data Science, Southern University of Science and Technology, Shenzhen 518055, China

**Keywords:** Multi-objective optimization, Evolutionary algorithm, Neural network pruning, Hardware-aware machine learning, Hardware efficiency

## Abstract

Neural network pruning is a popular approach to reducing the computational complexity of deep neural networks. In recent years, as growing evidence shows that conventional network pruning methods employ inappropriate proxy metrics, and as new types of hardware become increasingly available, hardware-aware network pruning that incorporates hardware characteristics in the loop of network pruning has gained growing attention. Both network accuracy and hardware efficiency (latency, memory consumption, etc.) are critical objectives to the success of network pruning, but the conflict between the multiple objectives makes it impossible to find a single optimal solution. Previous studies mostly convert the hardware-aware network pruning to optimization problems with a single objective. In this paper, we propose to solve the hardware-aware network pruning problem with Multi-Objective Evolutionary Algorithms (MOEAs). Specifically, we formulate the problem as a multi-objective optimization problem, and propose a novel memetic MOEA, namely HAMP, that combines an efficient portfolio-based selection and a surrogate-assisted local search, to solve it. Empirical studies demonstrate the potential of MOEAs in providing simultaneously a set of alternative solutions and the superiority of HAMP compared to the state-of-the-art hardware-aware network pruning method.

## Graphical Abstract

**Figure:**
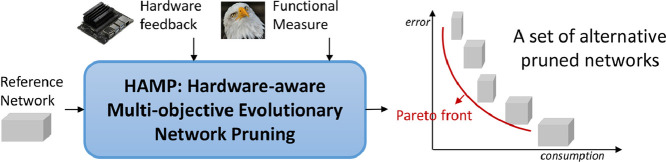

## 1 Introduction

Recent breakthroughs in applications such as image classification [1] and natural language processing [2] have brought the increased popularity of Deep Neural Networks (DNNs). However, the computationally expensive and memory-intensive properties of DNNs have hindered their development on resource-limited platforms such as smartphones and embedded sensors. To address this issue, various types of model compression techniques [3], [4], [5] have been proposed. Among them, network pruning has attracted extensive attention from both academia and industry.

Network pruning removes redundant parameters to generate a sub-network of the original DNN to reduce network complexity. In the past decade, network pruning methods have been proposed based on various proxy metrics of network complexity, such as the number of Multiply-Accumulate operations (MACs) [4], the number of FLoating-point Operations Per Second (FLOPS) [6], [7], [8], [9], [10], the number of connection weights [11], [12], [13], the number of blocks [14], etc. Despite their simplicity, two disadvantages of these proxy metrics have been raised recently. First, growing evidence has shown that these proxy metrics do not necessarily yield optimal DNNs in terms of hardware efficiency [15]. For instance, MobileNetV2 [16] has less than one-third of the number of parameters of ShuffleNet [17], but it consumes more than 1.1 times the memory of ShuffleNet. Second, hardware characteristics were not involved in the proxy metrics investigated before, while the latency, memory consumption, and energy consumption of a real-world DNN system could be highly hardware-dependent, due to the differences in hardware and software implementation of different hardware platforms.

For the above two disadvantages of proxy metrics, and with the emergence of a wide variety of smart chips, growing efforts are made to incorporate hardware characteristics in the loop of network pruning [18], referred to as hardware-aware neural network pruning in this paper. According to the access mode to hardware platforms, existing hardware-aware network pruning can be categorized as hardware estimation model-based [19], [20], [21], [22], [23] and direct feedback-based methods [24,25]. A hardware estimation model could be viewed as a proxy metric that involves hardware characteristics. It requires experts to construct analytic representations that well approximate target hardware platforms. To name a few, ECC [18] requires a differentiable bilinear energy estimation model to perform network pruning; AdaDeep [19] relies on a synchronous dataflow model-derived latency estimation model; EAP [20] is reliant on an energy estimation model based on data sparsity and bit-width reduction analysis. In contrast, direct feedback-based methods directly employ the real measurements of a DNN's hardware efficiency at inference time. For instance, the average forwarding time of one batch is used to measure the latency to facilitate network pruning [24], and a layer-wise lookup table with pre-measured latency of each layer is employed [25]. This type of measurements enables the direct feedback-based methods to be highly flexible and portable.

Despite these efforts, the current hardware-aware neural network pruning remains challenging due to two issues. First, although direct feedback offers potential for portability, it is typically black-box and non-differentiable, due to the complexity of computational units and memory access patterns. Second, both accuracy and hardware efficiency are important for a pruned network, and it is unrealistic to overlook either. However, all the methods mentioned above handle this as optimization problems with a single objective, i.e., optimize only one metric and convert all others to constraints [19,21,[23], [24], [25]], or aggregate the metrics into a scalar function [20,22]. Such an approach might be inappropriate because accuracy and hardware efficiency are generally in conflict and of different scales, which means a single solution is impossible to be optimal in both of them. Summing up metrics of different scales does not provide meaningful information about solution quality and may cause difficulties in practice as it is hard to determine appropriate weights.

Motivated by the above consideration, we propose to employ Multi-Objective Evolutionary Algorithms (MOEAs) to solve the hardware-aware neural network pruning problem. Compared with conventional optimization algorithms, MOEAs have two advantages in tackling this problem. One is that MOEAs do not require particular assumptions like differentiability or continuity and possess strong capacity for black-box optimization. The other is their ability to find multiple Pareto-optimal solutions in a single simulation run, which is very useful in practice because it offers flexibility to meet different user requirements. Specifically, once such a set of solutions has been found, the end users can easily choose their preferred configurations of DNN compression (e.g., latency first or memory consumption first) with just one click on the corresponding solutions. In the past few decades, MOEAs have drawn considerable attention and their success has been demonstrated on a large spectrum of real-world applications [26], [27], [28], [29]. However, to the best of our knowledge, the only work reported on hardware-aware network pruning based on MOEAs is our previous attempt [30]. Though this attempt implies some potential of MOEAs, it requires specialized acceleration mechanisms due to its unstructured pruning. Instead, this paper focuses on filter pruning to gain more compatibility with general-purpose hardware, and also improves upon the previous attempt to be capable of optimizing multiple hardware-based metrics. To this end, a novel MOEA named Hardware-Aware Multi-objective evolutionary network Pruning (HAMP) is proposed. HAMP can automatically search for a set of Pareto-optimal pruned networks that are well-adapted to the target hardware, and as will be shown by the experimental studies, HAMP can provide competitive performance in terms of both accuracy and hardware efficiency compared with the single-objective hardware-aware neural network pruning approach.

In summary, we make the following contributions in this paper:

1) In order to generate pruned networks that can be automatically adapted to hardware with trade-offs between accuracy and hardware efficiency, we formulate it as a multi-objective optimization problem. The problem, once solved, will deliver simultaneously a set of alternative solutions, which enables users to conveniently make their choices to meet specific demands.
2) We propose a novel MOEA, dubbed HAMP, to solve the problem. It is a memetic MOEA that combines an efficient portfolio-based selection and a surrogate-assist local search operator. HAMP is currently the only network pruning approach that can effectively handle multiple hardware direct feedbacks and accuracy simultaneously.
3) Experimental studies on the mobile NVIDIA Jetson Nano demonstrate the effectiveness of HAMP over the state-of-the-art and the potential of MOEAs for hardware-aware network pruning.

The remainder of this paper is organized as follows. Section 2 presents the problem formulation of the multi-objective hardware-aware network pruning. Section 3 introduces the evolutionary multi-objective optimization and proposes HAMP. Section 4 is devoted to experimental studies to evaluate the efficacy of HAMP. Finally, conclusions and discussions on future directions are presented in Section 5.

## 2 Problem formulation

Given a pre-trained DNN $\varphi_{0}$, one would like to find a pruned network φ that can consume as few hardware resources as possible while preserving the original task performance of $\varphi_{0}$ as closely as possible. However, the improvement of task performance typically leads to networks with higher complexity, which consumes more resources. It is thus impossible that a single pruned network exists that can be optimal for both accuracy and hardware efficiency. Instead, a set of compromise solutions (largely known as Pareto-optimal solutions) arises. Existing approaches often tackle this issue by transforming the metrics into a scalar function and solving the resultant single-objective optimization problem. In contrast, in this paper, we propose to simultaneously consider all metrics as objectives with multi-objective optimization and search for a set of well-distributed Pareto-optimal solutions.

The problem of the multi-objective hardware-aware network pruning can be expressed as follows. Suppose the pre-trained DNN $\varphi_{0}$ is a network with D layers to be pruned, where the ith layer contains $\theta_{i}$ filters, i=1,⋯,D. A solution of hardware-aware network pruning can be represented by a subset of filters of $\varphi_{0}$ to be reserved to generate a pruned network φ. However, given that the size of such a subset often changes during the search process, such a representation will result in solutions with variable length and introduce uncertainties to algorithm design. Alternatively, a Boolean vector that directly characterizes whether each filter is reserved or not can be of fixed length, which is often used in evolving neural networks [31]. The problem is that the current practice works mainly on small-scale networks, while modern DNNs usually contain many thousands of filters. In this paper, a fixed-length vector that indicates the proportion of filters to be reserved in each layer is adopted. Since the number of layers D is much smaller than the total number of filters $\sum_{i=1}^{D}\theta_{i}$, this approach can alleviate the above issues. More formally, a solution to hardware-aware network pruning is represented by $x=\{x_{1},x_{2},\cdots ,\;x_{D}\}$, where $x_{i}\in \left(0,1\right]$ indicates the proportion of filters to be reserved in the ith layer, i=1,⋯,D. For each $x_{i}$, the number of filters to be reserved is $\left\lceil \theta_{i}\cdot x_{i}\right\rceil$, where the function ⌈a⌉ returns the smallest integer larger than a. Then the specified number of filters with the largest l2-norm magnitude in each layer is reserved to generate a pruned network $\varphi_{x}$. Using the above solution representation, the multi-objective hardware-aware network pruning problem can be formulated as follows:

(1) $$\min F\left(x\right)=\left\{ \begin{array}{c} f_{0}\left(x\right)=1-Acc\left(\varphi_{x}\right)\\ f_{1}\left(x\right)=H_{1}\left(\varphi_{x}\right)\;\\ \cdots \;\\ f_{M}\left(x\right)=H_{M}\left(\varphi_{x}\right)\; \end{array}\right.$$

$$s.t.\;Acc\left(\varphi_{x}\right)\geq \epsilon \cdot Acc\left(\varphi_{0}\right)$$

The first objective function $f_{0}$ aims to maximize accuracy of a pruned network, which is also required to maintain its original accuracy as much as possible by a constraint ratio ϵ. The other M objective functions $f_{1},\cdots ,f_{M}$ require minimizing hardware overheads. They can be energy, power, latency, memory, etc. In this paper, we are interested in metrics that are important and can be obtained with direct feedback without requiring specialized detection equipment, which is common in practice. For this reason, latency and memory consumption are considered in this paper. Let $H_{1}\left(\varphi_{x}\right)=Lat\left(\varphi_{x}\right)$ and $H_{2}\left(\varphi_{x}\right)=Mem\left(\varphi_{x}\right)$ indicate the direct feedback of latency and memory consumption for network inference, respectively. These objective functions are typically discontinuous and non-differentiable due to complex memory access patterns and cache hierarchy involved in modern hardware.

Due to the conflicting nature of the objective functions of Eq. 1, we are concerned with finding a set of optimal trade-offs, the so-called Pareto-optimal solutions. The Pareto optimality concept [26] is formally defined as follows. A solution x is said to dominate a solution z, denoted as F(x)≺F(z), if and only if $\forall i\in \left\{0,\cdots ,M\right\}:f_{i}\left(x\right)\leq f_{i}\left(z\right)$ and $\exists j\in \left\{0,\cdots ,M\right\}:f_{j}\left(x\right)<f_{j}\left(z\right)$. Two solutions are related to each other in two possible ways: either one dominates the other or they are non-dominated. A solution $x^{*}$ is called a Pareto optimal solution if and only if $∄z\in \Omega :F\left(z\right)≺F\left(x^{*}\right)$, where Ω is the feasible region of problem Eq. 1. The set of all the Pareto-optimal solutions is called the Pareto set, and the image of the Pareto set in the objective space is called the Pareto front.

## 3 Hardware‐aware multi‐objective evolutionary network pruning

**Fig. 1:**
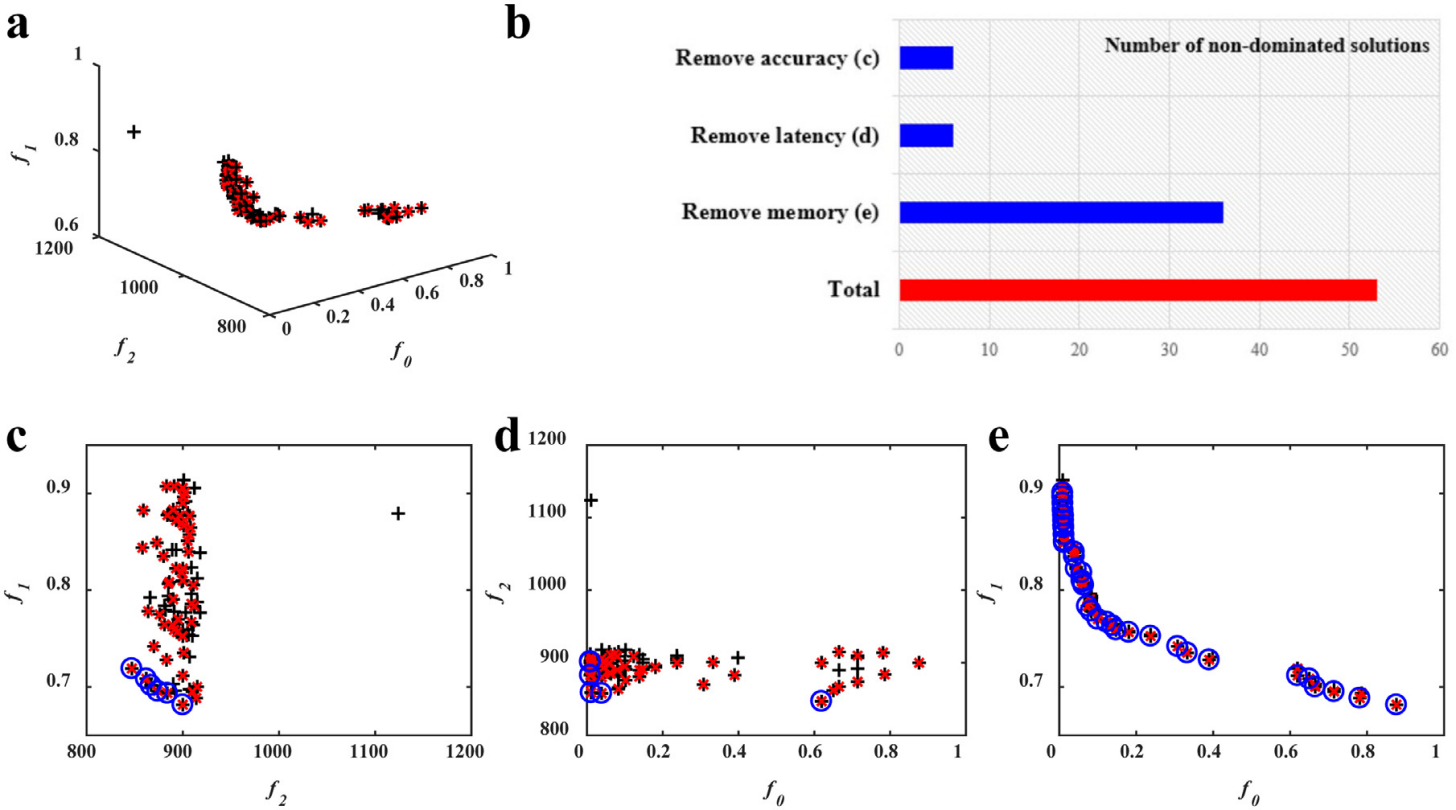
**Study of the change in the dominance structure brought about by the removal of an objective function**. (a) A set of randomly sampled solutions (black ‘+’) and its non-dominated solutions (red ‘×’). (b) The number of the retained non-dominated solutions after removing each objective function. (c) The retained non-dominated solutions (blue ‘o’) after removing $f_{0}$. (d) The retained non-dominated solutions (blue ‘o’) after removing $f_{1}$. (e) The retained non-dominated solutions (blue ‘o’) after removing $f_{2}$.

As mentioned in Section 2, the hardware-aware network pruning involves multiple, conflicting objectives, and the objective functions are often discontinuous and non-differentiable. These features cause difficulties to traditional optimization algorithms. Alternatively, evolutionary optimization has emerged as an effective approach to multi-objective black-box optimization and a large number of MOEAs have been proposed [26]. Among them, NSGA-II [32] has shown superior performance on not only benchmarks [33,34], but also real-world applications [35], and is one of the most popular MOEAs. For this reason, we first employed NSGA-II to tackle the hardware-aware network pruning problem. However, as will be shown in the experimental studies (Section 4.2), NSGA-II has difficulties in converging towards the Pareto front. We have investigated this issue in-depth and found that the failure of NSGA-II may be due to two features that will be detailed below and shown in Fig. 1. Motivated by this, the novel HAMP that integrates NSGA-II with two new operators is proposed. In the following, we will first present our preliminary studies, and then introduce the proposed HAMP.

### 3.1 Preliminary studies

This section aims to study the possible factors that may render NSGA-II ineffective for the hardware-aware network pruning problem. Through studying the objective functions of this problem as well as the relationship between them, we suppose that the inefficiency of NSGA-II may be due to the following two features.

The first is that the different impact of filter pruning and hardware on different objective functions may result in an imbalanced objective space that can be challenging to the non-dominated sorting approach employed in NSGA-II. The imbalance means that the change in the dominance structure brought about by the removal of an objective function is different. This is detailed in the following. The accuracy of a pruned network usually incurs significant deterioration as modern DNNs involve numerous highly nonlinear computations, while it does not vary across hardware. In contrast, some hardware-based metrics are likely to be less sensitive to pruning due to their dependence on hardware. Thus, it is possible that some objective functions behave in a much less conflicting manner. For instance, the study of resource mapping and layer accuracy impact of pruning on VGG shows that compared with the conflict between accuracy and latency or energy, accuracy is much less conflicting with memory [22]. The results examined on smartphones and smart home devices by combining compression techniques [20] implies that compared with the conflict between accuracy and latency, accuracy and energy are less conflicting. Our examinations on NVIDIA Jetson Nano also verify such phenomenon. Fig. 1 presents an example calculated on 100 pruned networks randomly sampled from AlexNet. The number of the non-dominated solutions still retained by removing each objective function is shown in Fig. 1b. It can be observed that compared to accuracy (Fig. 1c) and latency (Fig. 1d) that play a critical role in the dominance structure, memory consumption (Fig. 1e) has a relatively small impact. Thus, these objective functions may vary in the role they play in an algorithm's search efficiency, since the search efficiency depends largely on the selection operator that decides which solutions are preserved based on the objective functions.

The second is that the highly nonlinear nature of DNNs makes it difficult to generate feasible pruned networks with acceptable accuracy. This, coupled with the fact that the training of a DNN is extremely time consuming, typically taking several days or even months, leads to inefficiency in generating new solutions. This issue could be more severe when MOEAs are employed due to their stochastic nature. Generally, classical MOEAs often generate new solutions in a random way without much efforts to utilizing domain knowledge. Although such a randomization principle has been widely shown to be beneficial for MOEAs when dealing with benchmark problems, it is likely to result in inefficiency in generating feasible pruned networks and compromises the exploitation capabilities of an algorithm. As a result, it often requires a large number (e.g., hundreds of thousands or millions) of evaluations for classical MOEAs to find a set of good feasible solutions. In contrast, a relatively small number of evaluations is often allowed in practice due to the time-consuming DNN computation. As will be shown in the experimental studies (Section 4.2), the examined classical MOEAs could hardly find feasible pruned networks within the limited evaluation times. Thus, the commonly-used solution generators in classical MOEAs may not be the optimal strategy for hardware-aware neural network pruning. Though some efforts have been made to provide customized solution generators for pruning DNNs [12], due to the different solution representations, it is often difficult to directly apply a genetic operator developed for one representation to another. In addition, as they are not developed for hardware-aware neural network pruning, they do not necessarily guarantee good performance in this context.

### 3.2 The proposed HAMP

To overcome the above drawbacks, we propose two alternative approaches. First, instead of employing the non-dominated sorting approach, we propose a novel portfolio-based selection strategy. The main idea is to transform the imbalanced objective space into two subspaces and employ a separate selection principle in each subspace. Specifically, based on the above analysis on the difference of objective functions on the dominance structure, the first subspace is formed by the more conflicting objective functions with respect to accuracy and latency. As these objective functions largely determine the dominance structure, the selection principle in this subspace plays a critical role in algorithm performance. Thus, similar to NSGA-II, we employ the non-dominated ranking and crowding-distance assignment to rank candidate solutions. The difference is that the dominance relationship and crowding distance are not computed in the original objective space, but only in this subspace to emphasize convergence. The second subspace is formed by the less conflicting objective functions with respect to latency and memory consumption. Different from the former, the selection principle in this subspace focuses more on diversity preservation. Thus, it only considers non-dominated solutions in the candidate solutions, and the dominance relationship and crowding distance are computed only in this subspace. At last, the two selection principles are used together to generate a ranking for candidate solutions as follows. Given a set of candidate solutions, the number k of solutions to be selected, and a parameter η specifying the proportion of solutions selected in the first subspace, we will select ηk solutions in the first subspace and (1−η)k solutions in the second subspace. One can make a purposeful reconfiguration of optimization resources to enhance search efficiency by adjusting subspace settings. Additionally, to encourage feasible solutions, we also employ a simple but efficient constraint handling strategy. That is, the candidate solutions are first sorted in descending order of their accuracy, and then the feasible ones are further sorted using the above two selection principles.

Second, to overcome the computational overhead issue in generating new solutions, we propose the following surrogate-assisted scheme. Instead of calculating the precise accuracy, it adopts a low-precision but low-complexity surrogate to reflect the change of accuracy during the generation of new solutions. The reduction ratio of the number of weights, which has been widely used in the literature of network pruning and shown some association with accuracy [36], is used as the surrogate. Specifically, let $NW(\varphi_{x})$ denote the number of weights of the network corresponding to a solution x. Given solution x, a new solution $x^{\prime }$ generated from x is acceptable if and only if :

(2) $$NW\left(\varphi_{x^{\prime }}\right)\geq NW\left(\varphi_{x}\right)\cdot \frac{f_{0}\left(x\right)}{1-Acc\left(\varphi_{0}\right)}\;$$

In this way, a new solution is restricted by the minimum number of weights and thus the expensive overhead of precise accuracy computation can be avoided. The surrogate is then integrated to develop the Restricted Layer-wise Pruning (RLP) operator. The RLP operator is inspired by the popular layer-wise magnitude pruning [7,37] that directly utilizes the typical layered architecture of modern DNNs. It employs a heuristic that explores its neighborhood by pruning each layer of a given network individually. Specifically, given a parent solution, denoted as $x=(x_{1},x_{2},\cdots ,x_{D})$, the RLP operator explores its neighborhood by searching each dimension one by one with the following process. For the search in the ith dimension, the value of $x_{i}$ is gradually decreased until finding the last solution $x^{\prime }$ that satisfies the restriction Eq. 2. When the search of all D dimensions (which can be implemented in parallel) is completed, the union of these newly generated solutions forms the neighborhood population with respect to solution x. The advantage of the RLP operator is that it allows a natural partitioning of the DNN structure and enables an efficient local search with the surrogate-assist layer-wise pruning.

**Fig. 2:**
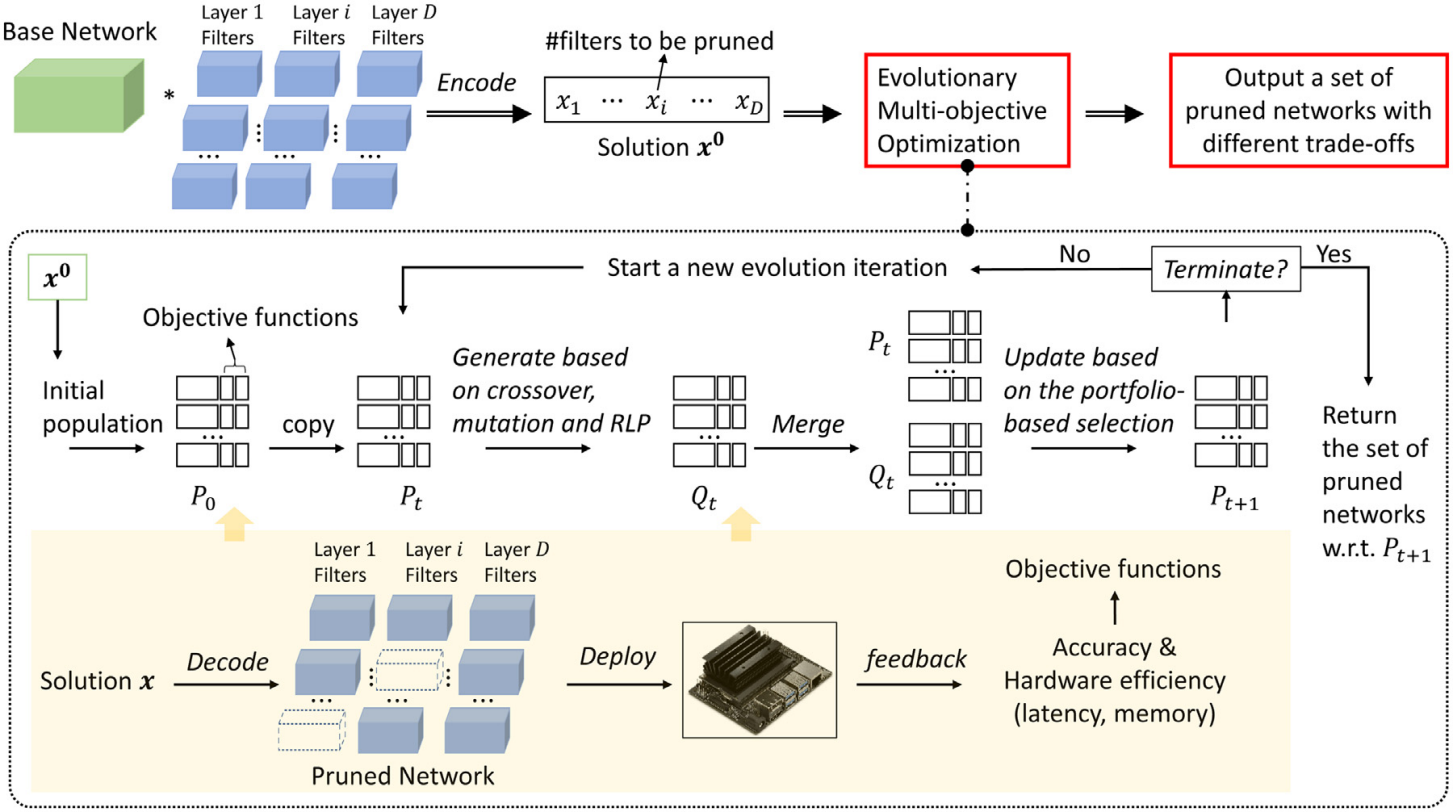
**The flowchart of the proposed HAMP**. It starts from the encoding of the base network (top left), iteratively searches for the set of Pareto-optimal pruned networks using evolutionary multi-objective optimization with reproduction and selection (middle), and outputs the pruned networks corresponding to the final population (top right). The quality of a solution is evaluated by its accuracy and direct hardware feedback (bottom).

Incorporating the above two operators into the framework of NSGA-II, we obtain a new MOEA, the HAMP, for the multi-objective hardware-aware network pruning. The pseudo-code of HAMP is shown in Algorithm 1 and its flowchart is presented in Fig. 2. Specifically, it conducts an iterative optimization by starting with a population of randomly initialized solutions, where a solution corresponding to the base network is also involved to ensure the existence of a feasible solution (lines 1-2), generating new solutions via classic genetic operators and the RLP operator alternately (lines 4-9), and selecting superior solutions to keep for the next generation based on the portfolio-based selection strategy (line 11). Taking advantage of its population-based nature and the two new operators, HAMP is able to deliver a set of solutions with diverse tradeoffs for flexible selection and is expected to converge better in the objective space than NSGA-II.

**Algorithm 1 HAMP**
**Input**: Base network $\varphi_{0}$, constraint ratio ϵ, population size n, iteration interval I.
**Output**: An approximation set P.
1.: Let $x^{0}=\left(1,\cdots ,1\right)$ denote the base network $\varphi_{0}$ and t=0;
2.: Initialize P with $x^{0}$ and n−1 solutions randomly generated based on $\varphi_{0}$;
3.: **while** stopping criterion is not met **do**
4.: **if** mod(t,I)=0 **then**
5.: Generate an offspring population Q with n solutions using crossover and mutation operators;
6.: **else**
7.: Randomly select a feasible solution x from P;
8.: Conduct a local search on x using the RLP operator and generate its neighborhood population Q;
9.: **end**
10.: Set R=P∪Q;
11.: Select n solutions from R using the portfolio-based selection operator;
12.: Update P to the set of the selected n solutions;
13.: Set t=t+1;
14.: **end**
15.: **return**P;

## 4 Experimental studies

Experimental studies have been carried out to evaluate the efficacy of our proposed approach. The experimental studies are designed to consist of three parts. The first study compares HAMP with the state-of-the-art hardware-aware neural network pruning method to evaluate the effectiveness of HAMP as well as whether HAMP is able to provide some advantage over the single-objective approach. Second, HAMP is compared with the representative MOEA. The third one investigates the efficacy of the two new operators for HAMP, respectively.

### 4.1 Experimental setup

Two well-known networks AlexNet [38] and MobileNet [9] are examined on a popular image classification dataset CIFAR-10. The training set contains 50,000 samples and the testing set contains 10,000 samples. The experimental platform consists of two parts. One is a typical hardware with limited resources used to deploy pruned networks, i.e., NVIDIA Jetson Nano with an integrated 128-core Maxwell GPU, quad-core ARM A57 CPUs, and 4GB memory. The other is a workstation used to run search algorithms, which contains four TITAN RTX GPUs and dual Intel Xeon Gold 6240 2.60GHz CPUs. The experiments are based on PyTorch with version 3.6.9.

When evaluating a pruned network, the direct feedback of latency and memory are used to measure its hardware efficiency. Specifically, the pruned network is examined on the NVIDIA Jetson Nano on a small set with a batch number of 10 and a batch size of 8, considering the limited resources of this mobile platform, and this examination is executed 10 times. The average running time in a single run is used as the latency and the peak of memory consumption during the whole process is used as the memory consumption. During the search, the accuracy of a pruned network is measured with a validation set with a batch number of 100 and a batch size of 128 is used. The accuracy constraint ratio is set to 0.9 for our intention to demonstrate the effectiveness of the algorithm on resource-limited platforms where a relatively high tolerance for accuracy is usually allowed. The accuracy of the final obtained pruned networks is evaluated on the testing set.

The six algorithms examined in the experiments are described as follows. The first is the state-of-the-art hardware-aware neural network pruning approach, namely NetAdapt [25]. NetAdapt is currently the only hardware-aware network pruning method that does not require specialized hardware or software support or certain hardware assumptions, while are not considered for their unstructured sparse networks [24] [30] that cannot be supported by off-the-shelf libraries [39]. For the settings, the recommended parameters provided in its released project are used, except that the lookup table is generated on Jetson Nano with a sample size of 10 and a batch size of 8, due to the limited computational resources of this mobile hardware, and the termination condition is set by the accuracy constraint. Two state-of-the-art non-hardware-aware network pruning methods are also involved to show the necessity of hardware-aware pruning approaches, i.e., White-Box [40] and AMC [41]. White-Box is a recently proposed method that has shown superior performance over a number of pruning methods, which employs FLOPs as the metric of network complexity. AMC is a representative pruning method that directly searches the redundancy for each layer, characterized by sparsity. For White-Box, the release project is used and extended to prune AlexNet and MobileNet. The pruning rate 0.3 is examined in the experiments and other parameters are set based on their default settings. For AMC, the release project is used and extended to prune MobileNet. AlexNet is omitted as the release code does not support its convolutional layer. The strategy search is set with its default settings and the fine-tuning is not involved for a fair comparison. The fourth is the representative NSGA-II, which is a dominate-based MOEA and one of the most popular MOEAs employed in academia and industry. The fifth is another popular MOEA, namely MOEA/D [42]. Different from NSGA-II, MOEA/D is a decomposition-based MOEA. The sixth is our proposed HAMP. The parameters of the MOEA-based approaches are set as follows. The dimensionality of the optimization task is 7 for AlexNet, and 14 for MobileNet. The population size is 10. The simulated binary crossover and polynomial mutation operator [43] are used and the probability of crossover and mutation is set to 0.9 and 0.1, respectively. The size of the offspring population generated by crossover and mutation is 10. The maximum objective evaluation time is 5,000. For MOEA/D, the number of neighbors is set to 5, the penalty-based boundary intersection approach is used for the decomposition, and uniform weight vectors are used. For HAMP, the proportion η in the portfolio-based selection scheme is 0.6. The iteration interval to use the RLP operator is 5. Each algorithm is executed for 10 independent runs.

The ablation study is also conducted by examining two variants of HAMP. One variant of HAMP is to validate the proposed portfolio-based selection operator, referred to as NPS-MOEA. In addition, the constraint handling strategy is retained in NPS-MOEA to better visualize the performance of the new selection operator in handling the imbalanced objective space. Thus, the difference between NPS-MOEA and HAMP lies in the portfolio-based selection operator. The two new selection principles (presented in Section 3.1) to deal with the imbalanced objective space are used in HAMP, while the classic non-dominated sorting and crowding-distance-based selection are used in NPS-MOEA. The other variant of HAMP is to validate the proposed RLP operator, referred to as NRLP-MOEA. The only difference between NRLP-MOEA and HAMP is the introduction of the RLP operator used to generate new solutions in HAMP.

### 4.2 Results and discussions

**Fig. 3:**
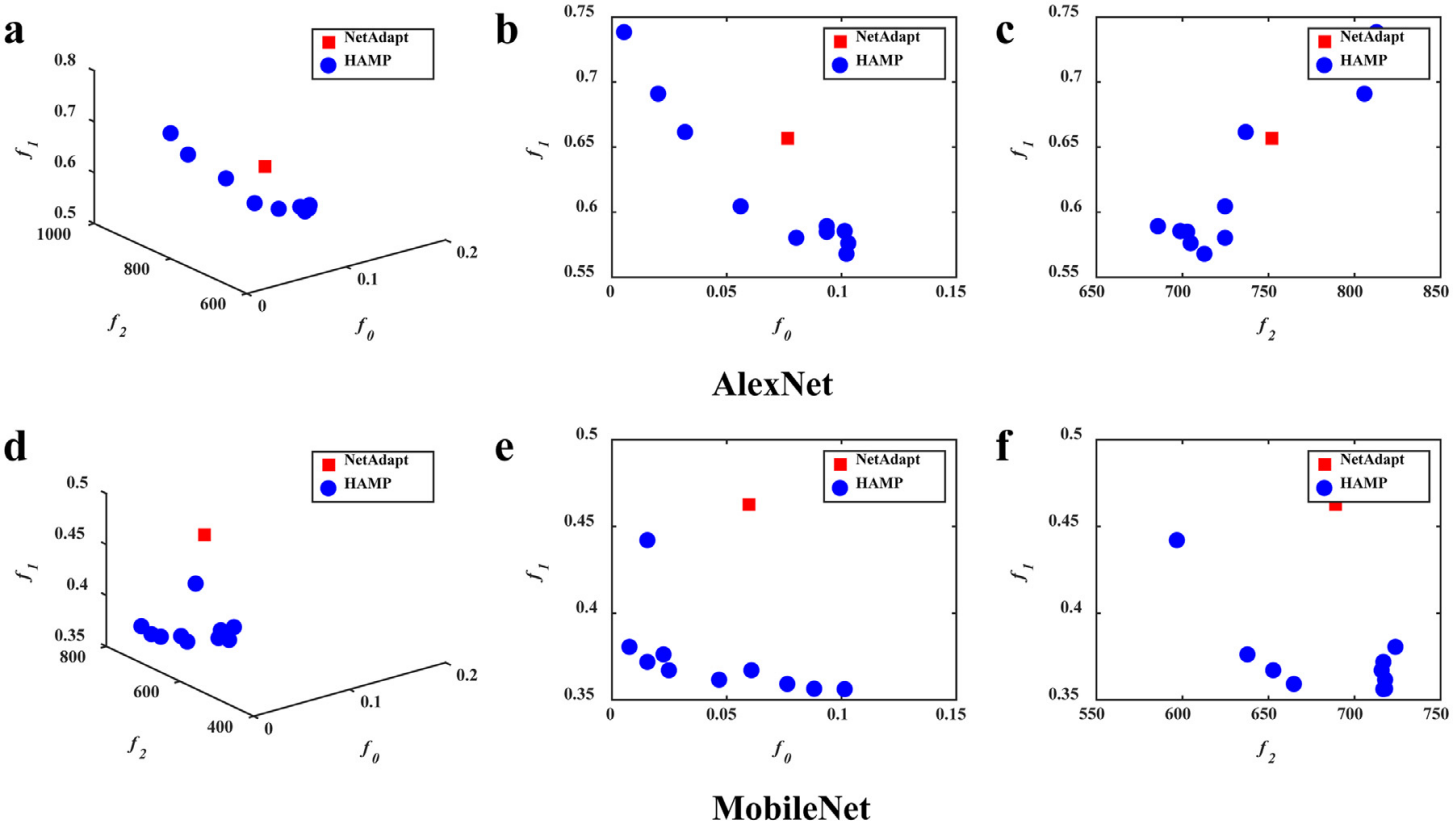
**The feasible solutions obtained by the algorithms**. HAMP not only manages to achieve solutions that are better than NetAdapt on all objectives, but also delivers simultaneously a set of alternative solutions.

**Fig. 4:**
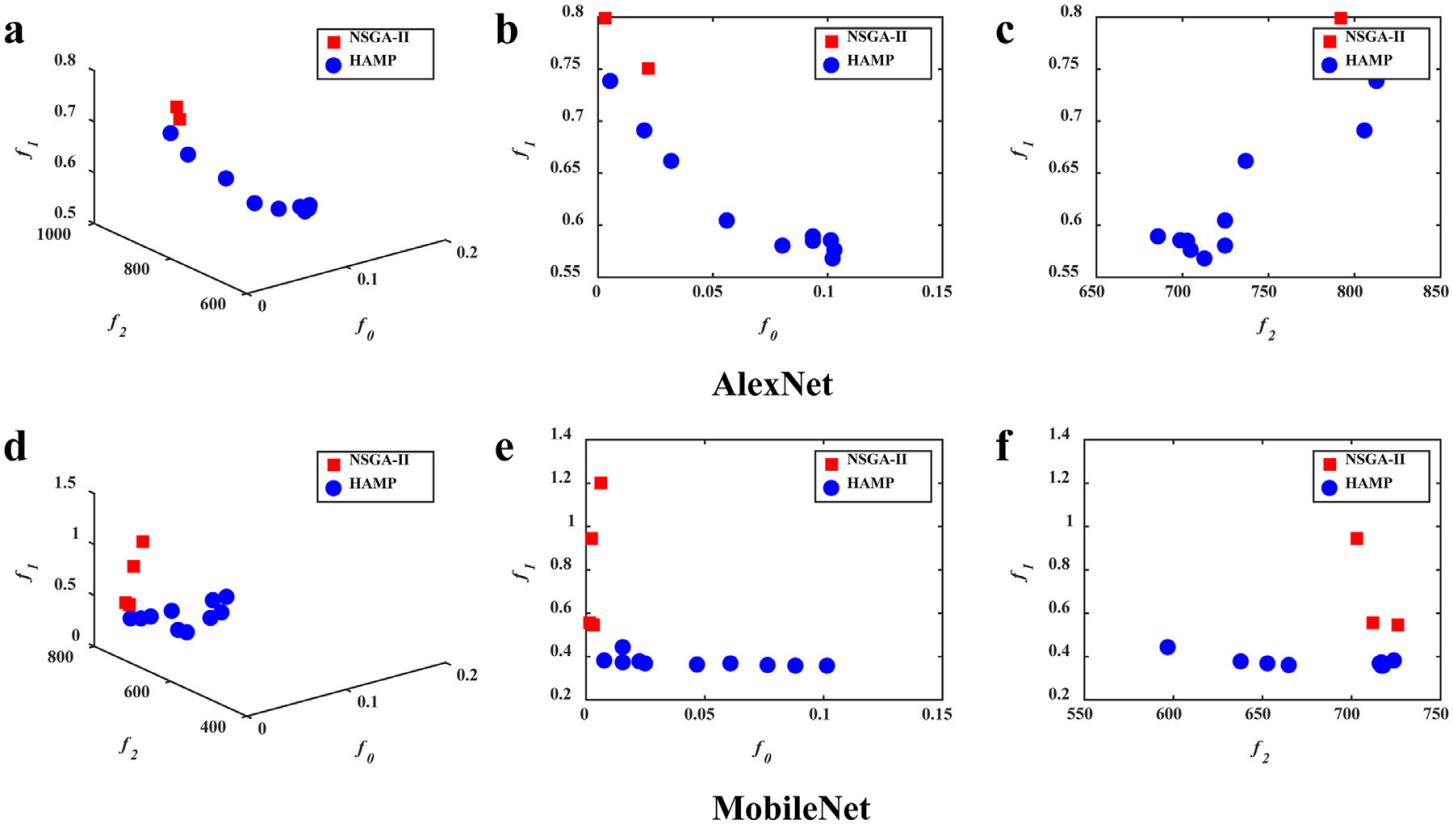
**The feasible solutions obtained by the algorithms**. HAMP yields more feasible solutions than NSGA-II and achieves smaller latency and memory consumption.

**Fig. 5:**
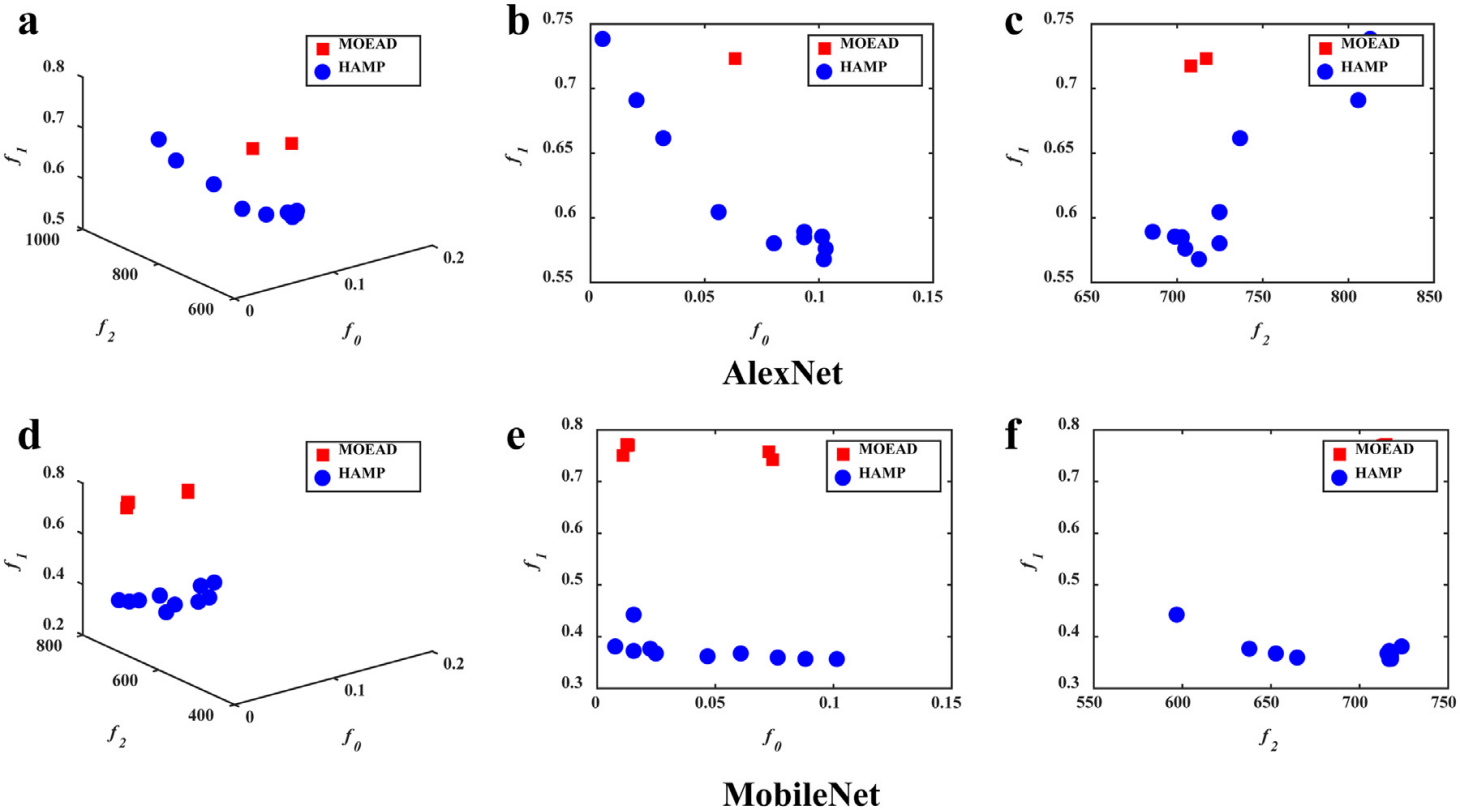
**The feasible solutions obtained by the algorithms**. HAMP yields more feasible solutions than MOEA/D and achieves smaller latency and memory consumption.

The feasible solutions obtained by HAMP and the baselines in the first simulation run in the objective space with respect to latency, memory consumption, and accuracy are illustrated in Fig. 3, Fig. 4, Fig. 5. The solution quality is further presented in a quantitative way in Table 1. In this table, the first thing should be noted is that, since MOEAs can yield a set of solutions in a single simulation run (as will be analyzed in detail below), multiple solutions are presented to show the ability of MOEA-based approaches in offering different trade-offs. We select some of them from the final set of feasible solutions and number them sequentially for ease of presentation, e.g., HAMP1/2/3/4 are referred to four solutions obtained by HAMP in the case of pruning AlexNet, and HAMP a/b/c/d are referred to four solutions obtained in the case of pruning MobileNet.

**Table 1:** **Solution quality comparison in terms of accuracy, latency and memory**.

	Latency (s)	Memory (MB)	RelValAcc (%)	RelTestAcc (%)
AlexNet	0.919	886	-	-
White-Box	0.806	894	10.3	11.0
NSGAII1	0.799	792	100.0	99.5
NSGAII2	0.751	833	98.2	98.0
HAMP1	0.738	813	99.8	99.3
MOEA/D1	0.723	717	94.0	93.9
MOEA/D2	0.718	708	90.4	90.0
NetAdapt	0.657	752	92.7	92.3
HAMP2	0.604	725	94.7	94.3
HAMP3	0.589	686	91.0	90.9
HAMP4	0.568	713	90.1	90.4
––––––––––––––––––––––––––––––––––––––––––––––––––––––––––––––				
MobileNet	1.476	801	-	-
NSGAII1	1.201	689	99.8	99.4
NSGAII2	0.945	703	100.0	99.8
White-Box	0.807	734	100.0	97.3
MOEA/D1	0.750	713	99.3	98.5
MOEA/D2	0.742	716	93.0	92.3
AMC	0.742	712	100.0	99.9
NSGAII3	0.546	726	100.0	99.8
NetAdapt	0.463	689	94.4	94.3
HAMPa	0.442	597	98.9	98.3
HAMPb	0.380	724	99.6	98.9
HAMPc	0.376	638	98.2	97.9
HAMPd	0.356	717	90.2	91.3

❖ The accuracy is shown by the relative accuracy compared to the base network, in terms of validate accuracy and testing accuracy. For each MOEA-based algorithm, a set of solutions are obtained, and we selected the solutions that can dominate as many previous results as possible from the generated solution set for comparison.

Generally, it can be observed that HAMP not only finds simultaneously a set of alternative solutions, but also manages to achieve better solutions that dominate the solution obtained by the baselines. First, the results in Table 1 show the good performance of the hardware-aware HAMP and NetAdapt over the non-hardware-aware White-Box and AMC in terms of latency and memory, implying the necessity of hardware-aware neural network pruning approaches. Second, from the illustrations in Fig. 3, Fig. 4, Fig. 5, it can be observed that compared with the single-objective NetAdapt, the three MOEAs are able to provide multiple solutions in a single simulation run. For example, as shown in Fig. 4, HAMP provides a set of solutions with their memory ranging from 686 MB to 833 MB, their latency ranging from 0.568 s to 0.751 s, and their relative test accuracy ranging from 90.0% to 99.8% when applied to AlexNet. Once such a set of solutions has been found by an MOEA, the end users can easily make a final decision by selecting their preferred options with respect to memory, latency, and accuracy (e.g., memory consumption first) with just one click on the corresponding solutions. This result suggests the high flexibility of MOEA-based approaches in providing a variety of trade-offs among latency, memory consumption and accuracy. The superiority of HAMP further demonstrates the competitiveness of MOEAs in solving hardware-aware network pruning. Third, when compared with NSGA-II and MOEA/D that do not perform satisfactorily and have difficulties in finding feasible solutions (suggested by the small number of feasible solutions illustrated in Fig. 4 and Fig. 5), HAMP is able to find more feasible solutions and the solutions it obtained spread better in the objective space. We do believe more in-depth analyses are required to study the efficacy of different MOEA frameworks as well as their variants in hardware-aware neural network pruning and we will leave these interesting problems for our future study.

The above analyses of the illustrations are further evaluated quantitatively, as shown in Table 1. Consistent with the above observations, HAMP yields significantly better solutions than the state-of-the-art hardware-aware network pruning NetAdapt in terms of all three objective functions, and it is better at generating pruned networks with lower latency and low memory consumption than NSGA-II and MOEA/D. For instance, as shown in Table 1, the solutions obtained by HAMP, i.e., HAMP2, HAMPa, and HAMPc, not only have smaller latency and consume less memory, but also have larger accuracy than that obtained by NetAdapt. In other words, the results of HAMP2, HAMPa, and HAMPc dominate that of NetAdapt. Furthermore, HAMP also provides other alternative solutions for user selection. HAMP3 is advantageous in memory consumption with a 22.6% reduction on AlexNet from 886 MB to 686 MB, while HAMP4 is superior in latency with a 38.2% reduction from 0.919 s to 0.568 s. Similarly, HAMPa achieves a 25.5% reduction in memory consumption and HAMPd gains a 75.9% reduction in latency on MobileNet. In summary, these results show that HAMP is superior to the baselines, offering not only a diverse set of solutions, but also competitive performance.

**Fig. 6:**
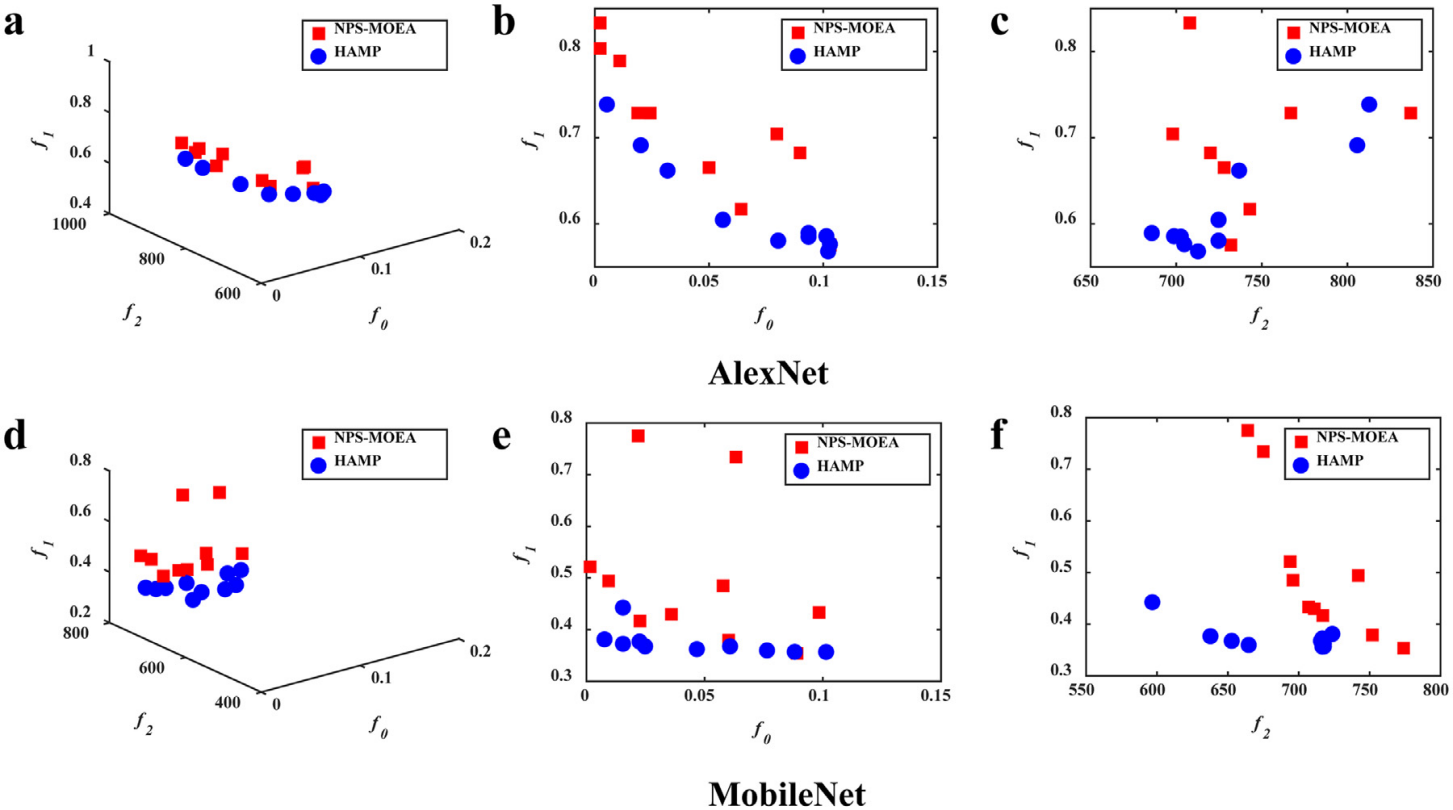
**The feasible solutions obtained by the algorithms**. HAMP converges better than NPS-MOEA, implying the utility of the portfolio-based selection operator.

**Fig. 7:**
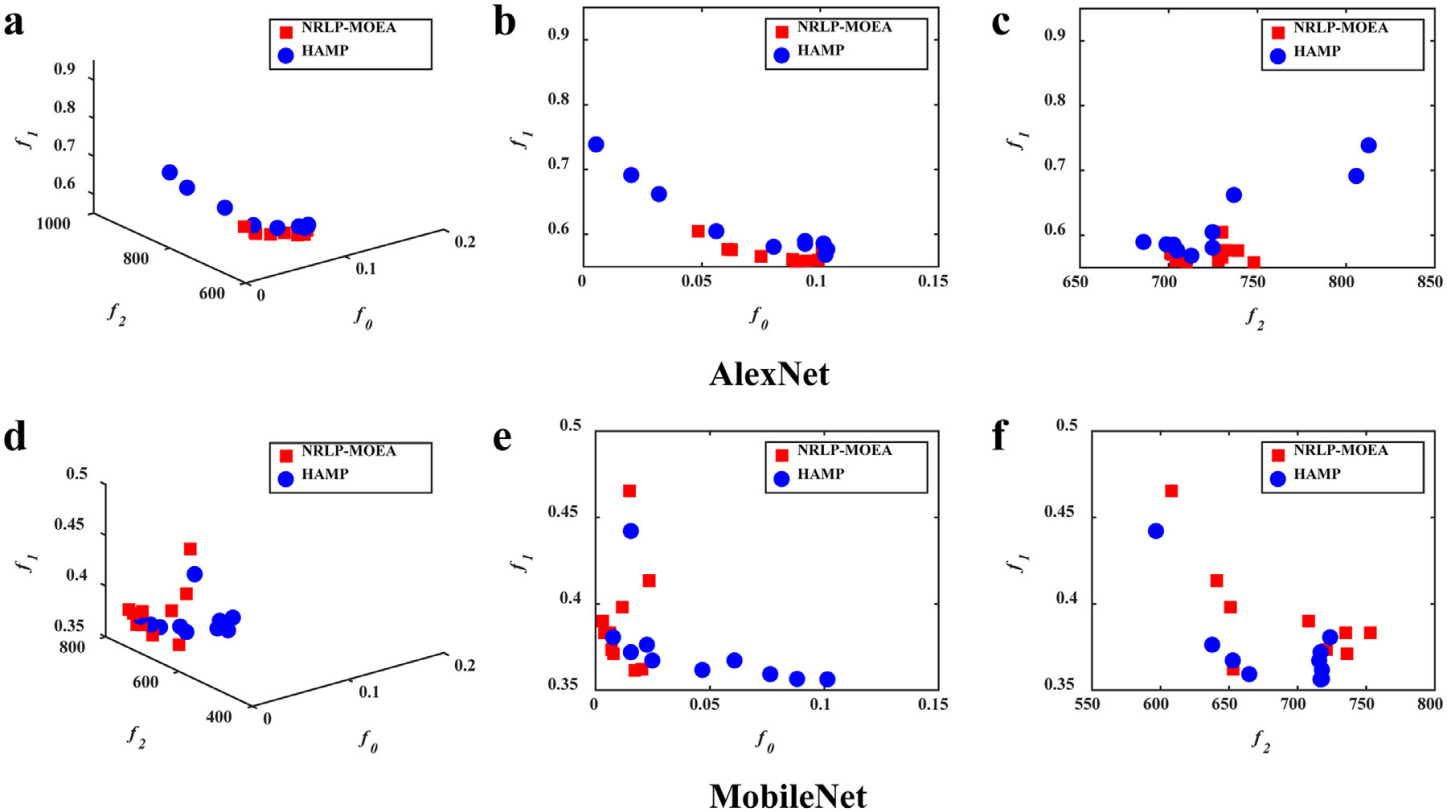
**The feasible solutions obtained by the algorithms**. HAMP spreads better than NRLP-MOEA, implying the utility of the RLP operator.

The comparisons between HAMP and its variants to evaluate the efficacy of the new operators are illustrated in Fig. 6 and Fig. 7. As shown in Fig. 6, HAMP achieves a better convergence towards the Pareto front compared with NPS-MOEA. This implies the utility of the portfolio-based selection in enhancing search efficiency for hardware-aware network pruning. Fig. 7 illustrates the results of HAMP and NRLP-MOEA. It can be observed that HAMP is much better in diversity, while NRLP-MOEA converges slightly better in some regions. This might be because the local search of the RLP operator involved in HAMP makes more search resources available for regions that have not yet been thoroughly searched. This allows solutions in these regions to have a chance to be improved and preserved for the next generation, leading to better diversity.

To further compare the MOEA-based approaches in a quantitative and rigorous way, we also adopt a quantitative metric to measure the quality of Pareto front approximations. In the multi-objective field, there are a number of quality indicators available for this purpose and among them, the hypervolume indicator [44] is of outstanding importance since it is sensitive to any type of improvements and it has been proved that the maximization of the hypervolume is equivalent to finding the Pareto set [45]. Thus, the hypervolume indicator is used in this experiment and the results are presented in Table 2. It is computed based on the normalized objective vectors of the non-dominated solutions, which is obtained by $f_{i}^{\prime }=\left(f_{i}-f_{i}^{min}\right)/\left(f_{i}^{max}-f_{i}^{min}\right)$, where $f_{i}^{min}$ and $f_{i}^{max}$ are the minimal and maximal values of $f_{i}$ among all the examinations, where i=0,⋯,M. The point (1.1,⋯,1.1) is used as the reference point since the values after the normalization always lie in the interval [0,1] and a slightly larger value for the reference point would be better [46]. The final hypervolume is normalized to the interval [0,1]. Statistical analysis with Wilcoxon rank-sum test [47] with a confidence level of 0.95 is also conducted. It can be observed that HAMP significantly outperforms the other MOEAs. This is consistent with the results presented in the previous discussions and further justifies the effectiveness of HAMP.

**Table 2:** **Comparison in terms of the hypervolume indicator (abbreviated as ‘HV’) for MOEA-based methods**.

	AlexNet		MobileNet	
Algorithm	Val HV	Test HV	Val HV	Test HV
MOEA/D	0.175*	0.178*	0.215*	0.207*
NSGA-II	0.109*	0.108*	0.347*	0.346*
NPS-MOEA	0.466*	0.483*	0.526*	0.515*
NRLP-MOEA	0.445*	0.457*	0.855*	0.816*
HAMP	0.537	0.538	0.868	0.842

❖ The larger the value, the better the result. The asterisk indicates that HAMP significantly outperforms the marked algorithm.

## 5 Conclusion

In this paper, we propose a multi-objective evolutionary optimization approach for the hardware-aware network pruning. We first formulate the hardware-aware network pruning as a multi-objective optimization problem to consider both direct hardware feedback and accuracy. Then, we solve the resultant problem using MOEAs. Specifically, a novel MOEA called HAMP is proposed because the popular MOEA, namely NSGA-II, does not perform satisfactorily on this problem. Experimental studies are carried out to evaluate the utility of HAMP in comparison with the state-of-the-art hardware-aware network pruning approach. The purpose is to demonstrate the potential of MOEAs in this field. The results show that HAMP not only manages to achieve solutions that are better on all objectives, but also delivers simultaneously a set of alternative solutions. These solutions present different trade-offs between latency, memory consumption, and accuracy, and hence can facilitate a rapid deployment of DNNs in practice.

There are several important future directions. The first is to apply the approach to other learning tasks, such as video tracking and natural language processing, to study the utility of MOEA-based hardware-aware neural network pruning methods on networks with different structures, sizes and parameters. Second, more sophisticated algorithm design towards the best performance under the proposed framework will be considered, e.g., investigating other types of MOEAs such as decomposition-based [42] and indicator-based MOEAs [44], and refining search strategies [48,49]. Besides, since currently the portfolio of selection principles is constructed and tuned manually, it is also interesting to investigate automatic approaches for constructing such principles [50], [51], [52] and theoretical performance [53]. Third, we are interested in further reducing the computational overhead of HAMP, which comes mainly from the evaluation of solutions, including the calculation of accuracy and the acquisition of direct hardware feedback (i.e., latency and memory).

## Declaration of competing interest

The authors declare that they have no conflicts of interest in this work.
